# Rationale, design, and baseline characteristics of a clinical trial for prevention of atherosclerosis in patients with insulin-treated type 2 diabetes mellitus using DPP-4 inhibitor: the Sitagliptin Preventive study of Intima-media thickness Evaluation (SPIKE)

**DOI:** 10.1186/1758-5996-6-35

**Published:** 2014-03-10

**Authors:** Tomoya Mita, Naoto Katakami, Toshihiko Shiraiwa, Hidenori Yoshii, Tomio Onuma, Nobuichi Kuribayashi, Takeshi Osonoi, Hideaki Kaneto, Keisuke Kosugi, Yutaka Umayahara, Tsunehiko Yamamoto, Kazunari Matsumoto, Hiroki Yokoyama, Mamiko Tsugawa, Masahiko Gosho, Iichiro Shimomura, Hirotaka Watada

**Affiliations:** 1 Department of Metabolism & Endocrinology, Juntendo University Graduate School of Medicine, Hongo 2-1-1, Bunkyo-ku, Tokyo 113-8421, Japan; 2 Department of Metabolic Medicine, Osaka University Graduate School of Medicine, 2-2, Yamadaoka, Suita, Osaka 565-0871, Japan; 3 Department of Metabolism and Atherosclerosis, Osaka University Graduate School of Medicine, 2-2, Yamadaoka, Suita, Osaka 565-0871, Japan; 4 Shiraiwa Medical clinic, 4-10-24 Houzenji, Kashiwara, Osaka 582-0005, Japan; 5 Department of Medicine, Diabetology & Endocrinology Juntendo Tokyo Koto Geriatric Medical Center, Shinsuna 3-3-20, Koto-ku, Tokyo 136-0075, Japan; 6 Misaki Naika Clinic, 6-44-9 Futawahigashi, Funabashi, Chiba 274-0805, Japan; 7 Naka Memorial Clinic, 745-5, Nakadai, Naka City, Ibaraki 311-0113, Japan; 8 Osaka Police Hospital, 10-31 Kitayamacho, Tennoji-ku, Osaka 543-0035, Japan; 9 Osaka General Medical Center, 3-1-56 Bandai-Higashi, Sumiyoshi-ku, Osaka 558-8558, Japan; 10 Kansai Rosai Hospital, 3-1-69 Inabasou, Amagasaki-shi, Hyogo 660-8511, Japan; 11 Diabetes Center, Sasebo Chuo Hospital, 15 Yamato-cho, Sasebo, Nagasaki 857-1195, Japan; 12 Jiyugaoka Medical Clinic, Internal Medicine, West 6, South 6-4-3, Obihiro 080-0016, Hokkaido, Japan; 13 Ikeda Municipal Hospital, 3-1-18, Jonan, Ikeda, Osaka 563-8510, Japan; 14 Unit of Biostatistics, Advanced Medical Research Center, Aichi Medical University, 1-1, Yazakokarimata, Nagakute, Aichi 480-1195, Japan

**Keywords:** Sitagliptin, DPP-4, Diabetes, Atherosclerosis, Intima-media thickness (IMT)

## Abstract

**Background:**

Sitagliptin, a dipeptidyl peptidase-4 (DPP-4) inhibitor, is currently used to achieve glycemic targets in patients with type 2 diabetes mellitus (T2DM). The addition of DPP-4 inhibitors to ongoing insulin therapy is expected to reduce insulin dosage, leading to a reduction in the frequency of hypoglycaemia and/or weight gain. Recent studies have demonstrated potential anti-atherosclerotic effects for DPP-4 inhibitors. The aim of the present ongoing study is to assess the effects of sitagliptin on the progression of atherosclerosis in patients with insulin-treated T2DM using carotid intima-media thickness (IMT), an established marker of cardiovascular disease.

**Methods and Design:**

The Sitagliptin Preventive study of Intima media thickness Evaluation (SPIKE) is a prospective, randomized, open-label, blinded-endpoint, multicenter, parallel-group, comparative study. Between February 2012 and September 2012, 282 participants who failed to achieve glycemic control despite insulin therapy were recruited at 12 clinics and randomly allocated to the sitagliptin group (n = 142) or the control group (n = 140). Primary outcomes are changes in maximum and mean IMT of the common carotid artery after 24-month treatment period measured by carotid arterial echography. Secondary outcomes include changes in glycemic control, parameters related to beta-cell function and diabetic nephropathy, occurrence of cardiovascular events and adverse events such as hypoglycaemia, and biochemical markers of vascular function.

**Discussion:**

The present study is designed to assess the effects of sitagliptin on the progression of carotid IMT. Results will be available in the near future, and the findings are expected to provide new strategy to prevent atherosclerosis in patients with insulin-treated T2DM.

**Clinical Trial Registration:**

UMIN000007396

## Background

Patients with type 2 diabetes mellitus (T2DM) are at high risk for cardiovascular disease (CVD), which are also the most frequent cause of death in these patients [1,2]. Thus, one of the main goals of T2DM management is to reduce the incidence of CVD. T2DM is a metabolic disorder characterized by a decline in insulin secretion and insulin resistance. To reduce blood glucose level, traditional oral hypoglycemic agents (OHA) have been widely used as complementary therapy. Although mono- and combination therapies temporarily improve glycemic control, it is often difficult to maintain long-term glycemic control. Eventually, many patients require insulin therapy in addition to OHA in order to achieve appropriate glycemic control.

Hypoglycaemia and weight gain are common side effects of treatment for T2DM [3] and the major barrier to achieving optimal glycaemic control, especially with insulin therapy. Indeed, strict glycaemic control using intensive insulin therapy increases the risk of hypoglycaemia threefold [4]. Also, weight gain by insulin therapy was widely observed in clinical studies [5-7]. Recent studies have been given questionable benefits of strict glycaemic control, especially using insulin, on CVD in patients with established atherosclerosis or longstanding T2DM [8-10], because frequent episodes of severe hypoglycaemia might reduce their beneficial effects [11] and weight gain may also adversely affect the prognosis. Therefore, several strategies should be employed in order to diminish these adverse effects of insulin therapy. One of the strategies is a reduction in insulin dose by stimulation of endogenous insulin secretion and increased insulin sensitivity using OHA. In fact, treatment with insulin plus metformin [12] and alpha glucosidase inhibitors [13], but not pioglitazone [5] or sulfonylurea [14], were advantageous in avoiding both weight gain and hypoglycaemia. However, there are treatment-limiting side effects for each specific drug. Alpha glucosidase inhibitors are associated with gastrointestinal symptoms including abdominal distension and flatulence [13]. The use of metformin is contraindicated in patients with renal or liver insufficiency and limited in the case of its gastrointestinal side effect [15]. Furthermore, the effects of treatment with insulin plus other traditional OHA on CVD prognosis remain largely unknown. Therefore, new OHA with the least risk of undeliverable effects and multiple beneficial effects on cardiovascular profiles when used with insulin are essential for the treatment of T2DM.

Sitagliptin, the first of a new class of dipeptidyl peptidase-4 (DPP-4) inhibitors, inhibits the degradation of active incretins by DPP-4. Sitagliptin high-selectively and reversibly inhibits DPP-4 compared to other members of the DPP family [16]. Sitagliptin produces approximately 2–3 fold increases in active glucagon-like peptide-1 (GLP-1) and glucose-dependent insulinotropic polypeptide (GIP) levels [16], which stimulates glucose-dependent insulin response [17-19]. GLP-1 decreases hepatic glucose output, glucagon release, gastric emptying, and appetite [20]. Also, GLP-1 seems to have a beneficial effect on functional pancreatic β cell mass [20]. Based on these properties, GLP-1 agonists can significantly decrease body weight, and DPP-4 inhibitors are considered weight neutral, both of which are advantageous relative to the weight gain seen with other OHA [20,21]. Indeed, the addition of sitagliptin to insulin therapy provided significant improvement in glycaemic control without increased risk of hypoglycaemia and clinically-relevant weight gain [22,23]. In addition, these novel agents have potential anti-atherosclerotic properties. GLP-1 directly acts on endothelial cells, vascular smooth muscle cells, monocytes, macrophages, and lymphocytes, and GLP-1 and GLP-1 receptor agonists have been shown to inhibit atherosclerosis and inflammation in rodents [24-26]. Likewise, DPP-4 inhibitors including sitagliptin also reportedly inhibit atherosclerosis and inflammation in both GLP-1-dependent and -independent manners [27,28]. Thus, the addition of DPP-4 inhibitors to insulin therapy is expected to have beneficial effects on CVD in patients with T2DM.

Two recent randomized clinical studies showed that DPP-4 inhibitors did not reduce the risk of CVD, but they also did not increase the risk compared to placebo in T2DM patients with a history of CVD or at risk for CVD [29,30]. These data may suggest that the potential effects of DPP-4 inhibitors in reducing CVD events rates was difficult to demonstrate especially after a relatively short period of treatment and because these subjects have already received a multitude of therapies for other pathologies, including statins, angiotensin inhibitors and antiplatelet agents. On the other hand, good glycemic control at an early stage of T2DM in patients free of history of CVD may increase the chance of significant reduction of not only microvascular disease but also CVD [31]. While early and effective intervention before the development of advanced atherosclerosis may be required to reduce the onset of CVD in CVD-free T2DM patients, it is no doubt difficult in clinical practice to assess the long-term effect of a single drug on primary CVD.

The carotid artery intima-media thickness (IMT) and its progression are considered a surrogate marker for CVD [32-34]. The marker has been widely used as a surrogate endpoint in the evaluation of the effects of intervention on the progression of atherosclerosis. To our knowledge, there are no published studies that have investigated the long-term anti-atherosclerotic effects of sitagliptin in insulin-treated T2DM patients. The present study is a multicenter, randomized, controlled trial designed to compare the effect of adding sitagliptin to insulin treatment on the progression of IMT in CVD-free T2DM patients (sitagliptin group), and sitaglitpin-untreated T2DM patients (control group).

## Methods and Design

### Study design

The Sitagliptin Prospective study of Intima media thickness Evaluation (SPIKE) trial is a prospective, randomized, open-label, blinded-endpoint, multicenter, parallel-group, comparative study. This study has been registered on the University Hospital Medical Information Network Clinical Trials Registry (UMIN-CTR), which is a non-profit organization in Japan and meets the requirements of the International Committee of Medical Journal Editors (ICMJE) (UMIN000007396).

### Study population

Japanese patients with T2DM who regularly attend the Outpatient Diabetes Clinics at 12 institutions in Japan are asked to participate in this study. The inclusion criteria are as follows: 1) T2DM patients in whom the target of blood glucose control specified in the Treatment Guide for Diabetes (Edited by Japan Diabetes Society) [35] was not achieved despite insulin therapy in addition to dietary/exercise therapy or concomitant therapeutic drugs for T2DM other than DPP-4 inhibitors over a period of 3 months or longer. Patients who withdrew from previous treatment with DPP-4 inhibitor for more than 12 weeks are included in the study, 2) ≥30 years of age or older and <80 years of age (regardless of gender), and 3) signing consent form for participation in the study. The following exclusion criteria are also applied: 1) type 1 or secondary diabetes, 2) presence of severe infectious disease, before or after surgery, or severe trauma, 3) history of myocardial infarction, angina pectoris, cerebral stroke, or cerebral infarction, 4) retinopathy requiring laser photocoagulation and/or vitrectomy, or history of these treatments within 1 year, 5) moderate or severe renal dysfunction (serum creatinine in mg/dL: males, >1.4; females, >1.2), 6) severe liver dysfunction (aspartate aminotransferase ≥100 IU/l), 7) moderate or severe heart failure (NYHA/New York Heart Association stage III or severer), 8) treatment with an incretin preparation, such as other DPP-4 inhibitors, at the start of the study, 9) treatment with drugs not concomitantly administrable with incretin preparations with regard to the national health insurance, such as DPP-4 inhibitors, at the start of the study, 10) pregnant, lactating, or possibly pregnant women, or those planning to become pregnant during the study period, 11) past medical history of hypersensitivity to investigational drugs, and 12) patients judged as ineligible by the clinical investigators.

The subjects are screened consecutively, and patients that meet the above eligibility criteria are asked to participate in the present study. All patients who agree to participate are entered into the study. The protocol was approved by the Institutional Review Board of each participating institution in compliance with the Declaration of Helsinki and current legal regulations in Japan. Written informed consent is obtained from all the participants after a full explanation of the study.

### Randomization and study intervention

Patients are registered at the administration office of the SPIKE trial via the internet, and once enrolled, they are randomly assigned to either the sitagliptin group or the control group on conventional treatment consisting of drugs other than the DPP-4 inhibitors. Randomization is performed using a dynamic allocation method based on the number of times of insulin injection, with/without pioglitazone, age, and gender.

Patients of the sitagliptin group are started on sitagliptin 25 mg once daily. The dose of sulfonylurea is tapered when considered clinically appropriate in order to avoid hypoglycaemia at the start of sitagliptin. Initiation of treatment with sitagliptin at 50 mg once daily is permitted in patients who are not treated with sulfonylurea. In patients treated with sitagliptin at 25 or 50 mg once daily for 12 weeks, the dose of sitagliptin is increased to a maximum dose of 100 mg once daily when HbA1c is ≥7.0% [35]. The participating physicians are allowed to reduce sitagliptin to 25 or 50 mg/day if treatment with 50 or 100 mg/day is not considered well tolerated. Insulin dose adjustment is also permitted, with priority given to achieve fasting blood glucose of <130 mg/dl and/or 2 hour postprandial blood glucose of <180 mg/dl, as recommended in the Treatment Guide for Diabetes [35]. In the control group, either increasing the dose of current therapy (e.g., insulin) or the addition of sulfonylurea, glinide and alpha glucosidase inhibitors is allowed with the goal of achieving the target value specified in the Treatment Guide for Diabetes (usually HbA1c level <6.9% and/or fasting blood glucose <130 mg/dl and/or 2 hour postprandial blood glucose < 180 mg/dl) [35]. The addition of other DPP-4 inhibitors and GLP-1 analogues is banned in the control group. The dose adjustment and addition of metformin and pioglitazone are banned in both groups during study.

**Figure 1:**
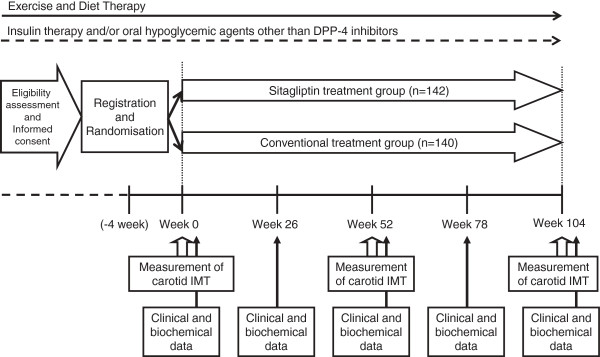
**Flow chart of the study schedule.** IMT; intima-media thickness.

In case of hypoglycaemia, the dose of insulin and/or OHA is titrated. Anti-hyperlipidemic and anti-hypertensive drugs are allowed to be used during the study (Figure 1).

### Observation variables and schedule

The observation parameters and schedule are shown in Table 1 and Figure 1. The study period is 2 years after registration of the patients (registration period: February 2012 to December 2013; full study duration: February 2012 to June 2016). All randomized participants will be followed until the end of the scheduled study, regardless of adherence to or discontinuation of study medication for any reason. Clinical outcome, adherence, and adverse events will be ascertained and clinical and biochemical data will be collected at 0, 26, 52, 78, and 104 weeks after randomization.

**Table 1:** Items and schedule

	**Registration**	**Treatment period (weeks)**					
	**visit 1 (-4 to 0 wks)**	**Visit 2 (0)**	**Visit 3 (26)**	**Visit 4 (52)**	**Visit 5 (78)**	**Visit 6 (104)**	**At discontinuation**
Patient characteristics	○						
Body weight	○	○	○	○	○	○	○
Blood pressure		○	○	○	○	○	○
Blood chemistry 1*		○	○	○	○	○	○
Blood chemistry 2^¶^		○		○		○	○
Urinary albumin excretion		○		○		○	○
Carotid IMT		○		○		○	○
baPWV and ABI^†^		○		○		○	○
Adherence			○	○	○	○	○
Adverse events		○	○	○	○	○	○

*Including aspirate aminotransferase, alanine aminotransferase, γ-glutamyl transpeptidase, creatinine, uric acid, total cholesterol, high-density lipoprotein cholesterol, low-density lipoprotein cholesterol, triglycerides, fasting plasma glucose, HbA1c and amylase.

^¶^Including C-peptide immunoreactivity, glucagon, serum intercellular adhesion molecule 1 (ICAM-1), vascular cell adhesion molecule 1 (VCAM-1), interleukin-6 (IL-6), high-sensitivity C-reactive protein (hs-CRP), remnant-like particle lipoprotein, 8-hydroxydeoxyguanosine (8-OHDG), thiobarbituric acid reactive substances (TBARS) and adiponectin.

^†^Including brachial-ankle pulse wave velocity (baPWV) and ankle brachial blood pressure index (ABI).

○Examinations were scheduled.

### Study outcome

The primary study outcomes are changes in maximum IMT of the right and left common carotid arteries (max-IMT-CCA) and mean IMT of the right and left CCA (mean-IMT-CCA) during the 24-month (=104 weeks) treatment period, as measured by carotid arterial echography. Investigations are carried out at the start of the study, after 12 months and after 24 months. The secondary outcomes are changes in 1) parameters related to glycemic control and β-cell function (HbA1c, fasting plasma glucose, C-peptide immunoreactivity, and glucagon), 2) parameters related to diabetic nephropathy, including urinary albumin excretion and estimated glomerular filtration rate, 3) lipid profile (total cholesterol, HDL-cholesterol, triglyceride, LDL-cholesterol, and remnant-like particle lipoprotein), 4) occurrence of cardiovascular events, including sudden death, coronary heart disease, and stroke, 5) hypoglycaemia and/or any other adverse events, and 6) changes in biochemical variables, including serum intercellular adhesion molecule 1 (ICAM-1), vascular cell adhesion molecule 1 (VCAM-1), interleukin 6 (IL-6), high-sensitivity C-reactive protein (hs-CRP), 8-hydroxydeoxyguanosine (8-OHDG), thiobarbituric acid reactive substances (TBARS) and adiponectin, 7) changes in treatment-related mental status, and 8) subsets of consenting patients in selected sites are enrolled in substudies designed to assess the effects of the intervention on brachial-ankle pulse wave velocity and ankle brachial blood pressure index.

### Safety evaluation

Details and incidence of adverse events are to be checked periodically.

### Measurement of carotid IMT

Ultrasonographic scans of the carotid artery are performed by expert sonographers who are specifically trained to perform the prescribed study examination, as reported previously [36]. To avoid inter-sonographer variability, each participant is examined by the same sonographer with the same equipment (high-resolution B-mode ultrasound scanner equipped with a high frequency (>7.5-MHz) linear transducer, with a limit of detection of <0.1 mm) throughout all the visits. The extracranial CCA, the carotid bulb (Bul), and the internal carotid artery (ICA) in the neck are scanned bilaterally in at least three different longitudinal projections (anterior, lateral, and posterior, which approximately corresponded to 60, 90, 150 degrees for the right carotid artery, and 210, 270, and 300 degrees for the left carotid artery marked on the Meijer’s Arc) as well as transverse projections, and the site of greatest thickness, including plaque lesions, is identified along the arterial walls. In this study, localized elevated lesions with maximum thickness of more than 1 mm, with a point of inflection on the surface of the intima-media complex, are defined as “carotid plaques”, based on the guideline of the Japan Society of Ultrasonics in Medicine [37]. IMT represents the distance between two parallel echogenic lines corresponding to the vascular lumen and the adventitial layer. To avoid inter-reader variability, all scans are electronically stored and emailed to the central office (IMT Evaluation Committee, Osaka, Japan) to be read by a single experienced reader blinded to the clinical characteristics of the patients, in a random order, using automated digital edge-detection software (Intimascope; MediaCross, Tokyo, Japan) [38]. The software system averages 60 points of IMT values in the segment 2 cm proximal to the dilation of the carotid bulb (mean-IMT-CCA). In addition, the greatest thicknesses of IMT, including plaque lesions in the CCA (max-IMT-CCA), the Bul (max-IMT-Bul), and the ICA (max-IMT-ICA), are also measured separately.

### Sample size

Yokoyama et al. [39] reported previously that the mean (±SD) rate of increase in carotid IMT in diabetic Japanese patients was 0.034 ± 0.054 mm/year and that 1% improvement in HbA1c was associated with 0.02 mm/year improvement in IMT. Based on these results, it is assumed that in a 2-year observation period, registration of at least 232 patients is required to obtain 80% power to detect a difference of 0.04 mm in IMT between the two treatment groups, assuming a standard deviation of 0.108, 15% dropout, and a 0.05 level of significance. Based on this calculation, the target number of enrolled patients is set at 274 for the 2-year registration period.

### Analysis population

With the exception of patients in whom IMT values are not measured at all during the observation period, data of all participants will be analyzed, regardless of their adherence to the study protocol, using an intent-to-treat approach (ITT).

### Safety and adverse events

For the sake of patient safety, all adverse events (AEs) would be recorded during the treatment and the follow-ups. AEs are defined as any untoward medical occurrence in a clinical trial subject administered a medicinal product and which does not necessarily have a causal relationship with this treatment.

The association between an AEs and the study medication must be classed as related or not related to the study drug by an investigator. All related AEs that results in a subject’s withdrawal from the study should be monitored until resolution. Serious AEs are defined as death or life-threatening events, which may require inpatient hospitalization, cause prolongation of existing hospitalization, or even result in persistent or significant disability/incapacity and need intervention to prevent permanent impairment or damage. If participants suffer any AEs/serious AEs, all details will be documented and reported. Furthermore, serious AEs will be reported to the principal investigator, the Data Safety Monitoring Board members (including two cardiologists and a neurologist) and the ethics committee. They can judge whether the diagnosis is appropriate or make a decision on whether the patient should withdraw from the trial based on reports.

### Statistical analysis

Efficacy will be analyzed using mainly the full analysis set based on the ITT principle and secondarily using the protocol set. To compare the change in IMT at Year 2 (Visit 6, representing the primary endpoint) relative to the baseline, statistical analysis is performed using unpaired *t*-test and analysis of covariance models that include treatment group, age, gender, baseline IMT, systolic blood pressure, and administration of statins. For the occurrence of cardiovascular events (representing a secondary endpoint), the time to the onset is analyzed and the event rate for each group is estimated using the Kaplan-Meier method and compared using the log-rank test. In addition, Cox proportional hazard model will be applied. The number and percentage of patients who develop adverse events is determined for each group and compared between the two groups using the Chi-square test. The level of significance is set at 0.05.

### Compliance with the Ethical Principles in Clinical Studies and Declaration of Helsinki

The study is to be conducted in accordance with the Ethical Principles in Clinical Studies published by the Ministry of Health, Labour and Welfare of Japan and the ethical principles originating in the Declaration of Helsinki.

### Trial organization

The SPIKE study was designed by the principle investigators (Hirotaka Watada, Department of Metabolism and Endocrinology, Juntendo University Graduate School of Medicine, Tokyo, Japan, and Iichiro Shimomura, Department of Metabolic Medicine, Osaka University Graduate School of Medicine, Osaka, Japan) through the SPIKE Project Office based at Soiken Inc., Chiyoda-ku, Tokyo, Japan. The principle investigators are responsible for all aspects of trial management, including collecting and cleaning all data, handling of all protocol-related issues, monitoring and optimizing adherence to interventions, adjudicating outcomes, auditing the progress of the study, and determining, executing, and publishing the final study analysis.

## Results

Between February 2012 and September 2012, 282 participants were recruited at 12 clinical sites and randomly allocated to either the sitagliptin group (n = 142) or the control group (n = 140). Eight patients were excluded from analyses due to withdrawal from participation. The baseline characteristics of the remaining 274 study participants are listed in Table 2. Of those randomized, 60.2% (n = 165) were male patients and the mean age was 63.7 years. The prevalence of hypertension was 58.8% and that of dyslipidemia was 63.5%. The mean fasting blood glucose level was 154 mg/dL and the mean HbA1c was 8.0%.

**Table 2:** Clinical characteristics of randomized participants in SPIKE

**Parameters**	
Gender (females/males) (%)	109 / 165 (39.8 / 60.2)
Age (years)	63.7 ± 9.8
Current smoking	59 (21.5)
Current alcohol	121 (44.2)
Hypertension	161 (58.8)
Dyslipidemia	174 (63.5)
Duration of diabetes (years)	17.3 ± 8.6
HbA1c (%)	8.0 ± 1.0
Fasting plasma glucose (mg/dl)	154.0 ± 48.9
C-peptide immunoreactivity (ng/ml)	1.2 ± 0.8
Glucagon (pg/ml)	80.5 ± 25.1
Body mass index (kg/m^2^)	25.0 ± 4.0
Systolic blood pressure (mmHg)	131.1 ± 15.0
Diastolic blood pressure (mmHg)	74.9 ± 11.5
Total-cholesterol (mg/dl)	192.5 ± 34.3
HDL-cholesterol (mg/dl)	55.1 ± 14.5
Triglyceride (mg/dl)	129.3 ± 100.7
LDL-cholesterol (mg/dl)	108.9 ± 28.7
Remnant-like particle lipoprotein (mg/dl)	5.1 ± 6.3
Serum creatinine (mg/dl)	0.7 ± 0.2
urinary albumin excretion (mg/g Cr)	19.3 [8.3-83.7]*
Serum uric acid (mg/dl)	5.3 ± 1.3
Amylase (U/l)	70.0 ± 34.5
Serum ICAM-1(ng/ml)	235.8 ± 90.9
Serum VCAM-1(ng/ml)	833.6 ± 340.3
IL-6 (pg/ml)	1.8 [1.2-2.9]*
hsCRP (mg/dl)	0.050 [0.024-0.116]*
Number of insulin injections	
<2/day	100
>3/day	174
Oral glucose-lowering agents	169 (61.7)
Metformin	97 (35.4)
Sulfonylurea	32 (11.7)
Glinides	21 (7.7)
Thiazolidinedones	24 (8.8)
α-glucosidase inhibitor	83 (30.3)
Anti-hypertensive drugs	147 (53.6)
ACE inhibitors	12 (4.4)
ARBs	122 (44.5)
Thiazides	25 (9.1)
CCB	84 (30.7)
Others	18 (6.6)
Lipid-lowering agents	145 (52.9)
Statins	129 (47.1)
Fibrates	6 (2.2)
Others	25 (9.1)
Anti-thrombotic agents	61 (22.3)
Antiplatelets	58 (21.2)
Anticoagulants	11 (4.0)

Data are number (%) or mean ± SD, or *median [range].

*ICAM-1*, Intercellular adhesion molecule 1; *VCAM-1*, Vascular cell adhesion molecule 1; *IL-6*, Interleukin 6; *hs-CRP*, High-sensitivity C-reactive protein; *ACE*, Angiotensin-converting enzyme; *ARB*, Angiotensin II receptor blocker; *CCB*, Calcium channel blocker.

## Discussion

The aim of this study is to investigate the effects of sitagliptin on the progression of atherosclerosis in insulin-treated patients with T2DM free of history of CVD, using common carotid IMT, a widely used surrogate marker of atherosclerosis, as the primary endpoint.

Controlling blood glucose with insulin is considered the most ideal therapy. However, there is still debate on whether insulin is actually beneficial in lowering CVD risk. Experimental studies have suggested that insulin may have beneficial as well as potentially harmful effects on the progression of atherosclerosis, as assessed by the expression of proinflammatory mediators and endothelial function [40-43]. Some observational studies reported a relationship between hyperinsulinemia and increased risk of CVD [44,45]. However, the Diabetes Control and Complications Trial/Epidemiology of Diabetes Intervention and Complication (DCCT/EDIC) study indicated that intensive treatment with insulin had long-term positive effects on CVD in patients with type 1 diabetes mellitus [46]. Similarly, multiple interventions that included insulin therapy, reduced the risk of CVD in the Steno study [47]. These data suggest that the use of insulin itself did not have deleterious effect on CVD prognosis. Nevertheless, recent studies questioned the benefits of strict glycaemic control, especially using insulin, on CVD in patients with established atherosclerosis or longstanding diabetes [8-10]. These might be largely affected by frequent episodes of severe hypoglycaemia [11] and weight gain with intensive insulin therapy. To reduce these negative aspects of insulin therapy, combination therapy of insulin plus other OHA might be one therapeutic option. The addition of DPP-4 inhibitors on ongoing insulin therapy was reported to have specific advantages on reduced frequency of hypoglycaemia and weight gain in addition to the expected benefits associated with glycemic control and limiting insulin dose [20,21]. Therefore, we expected that DPP-4 inhibitors added to on ongoing insulin therapy have beneficial effects on risk of CVD.

DPP-4 inhibitors may have unique direct beneficial effects on the progression of atherosclerosis in addition to the expected advantages described above in the case of co-administration with insulin. Experimental studies using animal models have shown anti-atherosclerotic effect for sitagliptin. For instance, Matsubara et al. [27] showed that inhabitation of macrophage inflammation by enhancing GLP-1 signalling with sitagliptin reduced atherosclerotic lesion formation in apoE-deficient mice. In addition, we and others recently demonstrated that other kinds of DPP-4 inhibitors also reduce the plaque burden at the level of the aortic sinuses accompanied by macrophage infiltration in animal model of atherosclerosis [28,48]. Furthermore, studies in rats demonstrated that treatment with sitagliptin reduced neointimal formation at 4 weeks after arterial injury [49]. These data suggest that DPP-4 inhibitors may have unique anti-atherosclerotic effects in patients with T2DM. Indeed, it was demonstrated that sitagliptin reduced monocyte inflammation in patients with T2DM independent of its glucose lowering effect [50]. Another study showed that sitagliptin improved endothelial dysfunction and inflammation in subjects with CVD and uncontrolled T2DM beyond its glucose lowering effect [51]. These effects may be mediated by blockade of degradation of a direct substrate of DPP-4, stromal cell-derived factor-1 (SDF-1), which is a chemokine known to stimulate bone marrow mobilization of endothelial progenitor cells (EPCs). In this regard, one recent study suggested that sitagliptin increased the number of circulating EPCs in T2DM patients with up-regulation of serum SDF-1, potentially leading to reduced progression of atherosclerosis [52]. Taken together, the results of the above preclinical and clinical studies have yielded new mechanistic insight and provided support to the beneficial effects of sitagliptin on CVD risk.

In contrast, two recent randomized short-term clinical studies showed that DPP-4 inhibitors neither reduced nor increased the risk of CVD compared to placebo in T2DM patients with history of CVD or at high risk for CVD [29,30]. With regard to sitagliptin, the randomized, placebo-controlled Trial Evaluating Cardiovascular Outcomes with Sitagliptin (TECOS) study has already commenced evaluation of the effects of sitagliptin on CVD in 14,000 patients with T2DM with longer duration of study period than other studies [53]. This study may provide further insight into the effects of DPP-4 inhibitors on prevention of CVD.

Recently, it was reported that short-term treatment with both sitagliptin and vildagliptin reduced the progression of IMT in subanalysis of a small number patients without a control group [54]. However, the results are probably of limited value due to the study design. On the other hand, the present study is designed to clarify the efficacy and benefits of sitagliptin in preventing the progression of atherosclerosis in patients with insulin-treated T2DM free of apparent CVD in a multicenter PROBE trial. We used surrogate endpoints in this trial due to practical constraints, including trial costs and concern about feasibility in relation to long-term intervention. Carotid ultrasonographic measurements of IMT have been validated against pathologic specimens, and were demonstrated to be strong predictors of CVD in subjects with and without T2DM [27,28]. It has also been shown that changes in carotid IMT over time correlate with the rate of future CVD [34].

The results will become available in the near future, and the findings might hold great clinical relevance to the prevention of atherosclerosis and subsequent CVD in patient with insulin-treated T2DM. The results from this clinical trial will be submitted for publication in 2014.

## Abbreviations

Bul: Bulb; CCA: Common carotid artery; CVD: Cardiovascular disease; DCCT/EDIC: Diabetes control and complications trial/epidemiology of diabetes intervention and complication; EPCs: Endothelial progenitor cells; GLP-1: Glucagon-like peptide-1; GIP: Glucose-dependent insulinotropic polypeptide; hs-CRP: High-sensitivity C-reactive protein; ICA: Internal carotid artery; ICMJE: International Committee of Medical Journal Editors; ICAM-1: Intercellular adhesion molecule 1; IL-6: Interleukin 6; IMT: Intima-media thickness; ITT: Intent-to-treat approach; T2DM: Type 2 diabetes mellitus; OHA: Oral hypoglycemic agents; 8-OHDG: 8-hydroxydeoxyguanosine; PROBE: Prospective, randomized, open label, blinded-endopoint; SDF-1: Stromal cell-derived factor-1; SPIKE: Sitagliptin Prospective study of Intima media thickness Evaluation; TBARS: Thiobarbituric acid reactive substances; TECOS: Trial Evaluating Cardiovascular Outcomes with Sitagliptin; UMIN-CTR: University Hospital Medical Information Network Clinical Trials Registry; VCAM-1: Vascular cell adhesion molecule 1.

## Competing interest

TM received research funds from MSD, Takeda and Eli Lilly. NKa is a staff member of the endowed chair (the Deapartment of Metabolism and Atherosclerosis) donated from Kowa Pharmaceutical Co. Ltd., has received lecture fees from Astellas Pharma Inc., AstraZeneca K.K., Daiichi Sankyo Inc., Dainippon Sumitomo Pharma Co., Eli Lilly, Mitsubishi Tanabe Pharma Co., Mochida Pharmaceutical Co., MSD, Novartis Pharmaceuticals, Novo Nordisk Pharma, Ono Pharmaceutical Co., Otsuka Pharmaceutical, Shionogi& Co., Ltd., Takeda Pharmaceutical Co., Teijin Pharma, Sanofi-Aventis. NKu has received lecture fees from Sanofi-Aventis, Eli Lilly. TO has received lecture fees from Boehringer Ingelheim, Sanofi-Aventis, Ono Pharmaceutical Co., Novo Nordisk Pharma, Kissei Pharma, Mitsubishi Tanabe Pharma, Novartis Pharmaceuticals, Sanwakagaku Kenkyusho, Daiichi Sankyo Inc., Takeda Pharmaceutical Co., MSD, Dainippon Sumitomo Pharm., Kowa Co. and research funds from Novo Nordisk Pharma, Dainippon Sumitomo Pharma. HK has received lecture fees from Boehringer Ingelheim, Sanofi-Aventis, Ono Pharmaceutical Co., MSD, Novo Nordisk Pharma, Novartis Pharmaceuticals, Daiichi Sankyo Inc., Takeda Pharmaceutical Co., Kissei Pharmaceutical Co., Dainippon Sumitomo Pharma Co., Mitsubishi Tanabe Pharma Co., Kyowa Kirin, and research funds from Takeda Pharmaceutical Co., MSD, Mochida Pharmaceutical Co. Sanofi-Aventis, Novartis Pharmaceuticals, Novo Nordisk Pharma, Eli Lilly, Daiichi Sankyo Inc., Shionogi Pharma, Teijin Pharma, Dainippon Sumitomo Pharma Co., Otsuka Pharmaceutical, Kissei Pharmaceutical Co. KK has received lecture fees from Boehringer Ingelheim, Sanofi-Aventis, Novo Nordisk Pharma, Novartis Pharmaceuticals, Eli Lilly, Takeda Pharmaceutical Co., MSD, Kowa Co, Mitsubishi Tanabe Pharma and research funds from Sysmex Co.. HYok has received lecture fees from Boehringer Ingelheim, Sanofi-Aventis, Ono Pharmaceutical Co., Novo Nordisk Pharma, Novartis Pharmaceuticals, Sanwakagaku Kenkyusho, Daiichi Sankyo Inc., Takeda Pharmaceutical Co., MSD, Dainippon Sumitomo Pharm., Kowa Co. and research funds from Sanofi-Aventis, Novo Nordisk Pharma, Novartis Pharmaceuticals, Sanwakagaku Kenkyusho, Takeda Pharmaceutical Co., MSD. MT has received lecture fees from Novartis Pharmaceuticals, Sanofi-Aventis, Kyowa Hakko Kirin Co., Novo Nordisk Pharma, Kowa Co. MG received lecture fees from Novartis. IS has received lecture fees from Astellas Pharma Inc., AstraZeneca K.K., MSD K.K., Ono Pharmaceutical Co., Ltd., Kyowa Hakko Kirin Co., Ltd., Kowa Pharmaceutical Co. Ltd., Sanofi K.K., Sanwa Kagaku Kenkyusho Co., Ltd., Daiichi Sankyo Co., Ltd., Takeda Pharma K.K., Mitsubishi Tanabe Pharma Co., Teijin Pharma Ltd., Eli Lilly Japan K.K., Nippon Boehringer Ingelheim Co., Ltd., Novartis Pharma K.K., Novo Nordisk Pharma Ltd., Bayer Yakuhin Ltd., Pfizer Japan Inc., Bristol-Myers K.K., Mochida Pharmaceutical Co., Ltd., and research funds from Astellas Pharma Inc., AstraZeneca K.K., Eisai Co., Ltd., MSD K.K, Otsuka Pharmaceutical Co., Ltd., Ono Pharmaceutical Co.,Ltd, Kaken Pharmaceutical Co., Ltd, Kissei Pharmaceutical Co.,Ltd, Kyowa Hakko Kirin Co., Ltd., Sanofi K.K., Shionogi & Co., Ltd., Daiichi Sankyo Co.,Ltd., Dainippon Sumitomo Pharma Co.,Ltd., Takeda Pharma K.K., Mitsubishi Tanabe Pharma Co., Teijin Pharma Ltd., Nippon Boehringer Ingelheim Co.Ltd, Novartis Pharma K.K., Novo Nordisk Pharma Ltd., Pfizer Japan Inc., Bristol-Myers K.K., Mochida Pharmaceutical Co., Ltd., Eli Lilly Japan K.K. HW has received lecture fees from Boehringer Ingelheim, Sanofi-Aventis, Ono Pharmaceutical Co., Novo Nordisk Pharma, Novartis Pharmaceuticals, Eli Lilly, Sanwakagaku Kenkyusho, Daiichi Sankyo Inc., Takeda Pharmaceutical Co., MSD, Dainippon Sumitomo Pharm., Kowa Co. and research funds from Boehringer Ingelheim, Pfizer, Mochida Pharmaceutical Co., Sanofi-Aventis, Novo Nordisk Pharma, Novartis Pharmaceuticals, Sanwakagaku Kenkyusho, Terumo Corp. Eli Lilly, Mitsubishi Tanabe Pharma, Daiichi Sankyo Inc., Takeda Pharmaceutical Co., MSD, Shionogi, Pharma, Dainippon Sumitomo Pharma, Kissei Pharma, and Astrazeneca.

## Authors’ contributions

The authors meet the criteria for authorship recommended by the International Committee of Medical Journal Editors (ICMJE) and take full responsibility for all contents of the manuscript and editorial decisions. All authors contribute to the study design and were involved at all stages of manuscript development. TM and NKa drafted the manuscript based on the protocol. MG contributes to analysis of research data. All authors were involved in analysis and interpretation of data, reviewed/edited the manuscript and approved the final manuscript. IS and HW were the principal guarantors of this work and have full access to all the data in the study and take responsibility for the integrity of the data and accuracy of data analysis.

## Authors’ information

Collaborators on Sitagliptin Preventive study of Intima media thickness Evaluation (SPIKE) Trial**:** (listed in alphabetical order)

Jiyugaoka Medical Clinic:

Hiroki Yokoyama

Juntendo Tokyo Koto Geriatric Medical Center (Department of Medicine, Diabetology & Endocrinology):

Kanae Ishida, Noriko Inagaki, Tomio Onuma, Keiko Yamashiro, Junko Yokota, Hidenori Yoshii

Juntendo University Graduate School of Medicine (Department of Metabolism & Endocrinology):

Fuki Ikeda, Koji Komiya, Tomoya Mita, Yuko Sakurai, Motoyuki Tamaki, Hirotaka Watada

Kansai Rosai Hospital:

Daisuke Azuma, Isao Hayashi, Isao Hayashi, Tsunehiko Yamamoto

Misaki Naika Clinic:

Nobuichi Kuribayashi

Naka Memorial Clinic:

Hidenori Isida, Takeshi Osonoi, Miyoko Saito

Osaka General Medical Center:

Masahiro Hatazaki, Ryutaro Kataoka, Yutaka Umayahara

Osaka Police Hospital:

Keisuke Kosugi, Ken’ya Sakamoto, Kazutomi Yoshiuchi

Osaka University Graduate School of Medicine (Department of Metabolic Medicine):

Hideaki Kaneto, Naoto Katakami, Taka-aki Matsuoka, Ken Ohya, Iichiro Shimomura, Sae Uno

Sasebo Chuo Hospital

Kazunari Matsumoto, Fumi Mori, Yoshitaka Mori

Shiraiwa medical clinic:

Toshihiko Shiraiwa

## Founding sources: (listed in alphabetical order)

Financial support for this study was provided by the Japan Society for Patient Reported Outcome, which is a general incorporated association. The Japan Society for Patient Reported Outcome received research funds from Mitsubishi Tanabe, Ono and Novo Nordisk.
